# The Zhang Knot: A Locking Slip‐Knot for Arthroscopy

**DOI:** 10.1002/atn2.70108

**Published:** 2026-05-12

**Authors:** Xingzhi Zhang, Zhenhua Li, Tao Zhang

**Affiliations:** ^1^ Class 19, Grade 2024 Shandong Experimental High School Jinan Shandong China; ^2^ Department of Cardiology The Central Hospital Affiliated to Shandong First Medical University Jinan Shandong China; ^3^ Department of Joint surgery and Sports Medicine The Central Hospital Affiliated to Shandong First Medical University Jinan Shandong China

## Abstract

The Zhang knot is a sliding knot for arthroscopic surgery that is simple to learn, easy to slide, and can be securely locked. This technique, which is similar to a reverse Tennessee knot, is described in detail with step‐by‐step illustrations and a video demonstration. Biomechanically, the Zhang knot provides strong initial holding strength and resists early slippage, allowing the surgeon to maintain desired tension before final locking. Its simple configuration, reproducibility, and minimal profile make it suitable for a variety of arthroscopic procedures. Overall, the Zhang knot represents a reliable and effective addition to the repertoire of arthroscopic knot tying techniques.

## Graphical abstract

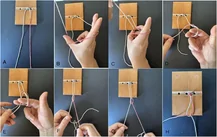

**VIDEO 1:** Demonstration of the Zhang sliding knot operation. Video content can be viewed at https://doi.org/10.1002/atn2.70108.

Secure arthroscopic knot tying is a fundamental skill for arthroscopic surgeons.^1^, ^2^, ^3^, ^4^ Typically, arthroscopic knot tying begins with a slip knot, followed by a series of half‐hitches to lock the knot.^5^ An ideal arthroscopic knot should slide easily, maintain tension without slack, and provide reliable security.

Most slip knots can slide in either direction, requiring careful handling to prevent loss of tissue hold while adding locking hitches.^6^, ^7^ Here, we describe a locking slip‐knot, the Zhang knot, which resembles a reverse Tennessee knot. This knot has a reproducible, simple configuration, and a minimal profile.

## TECHNIQUE

**FIGURE 1:**
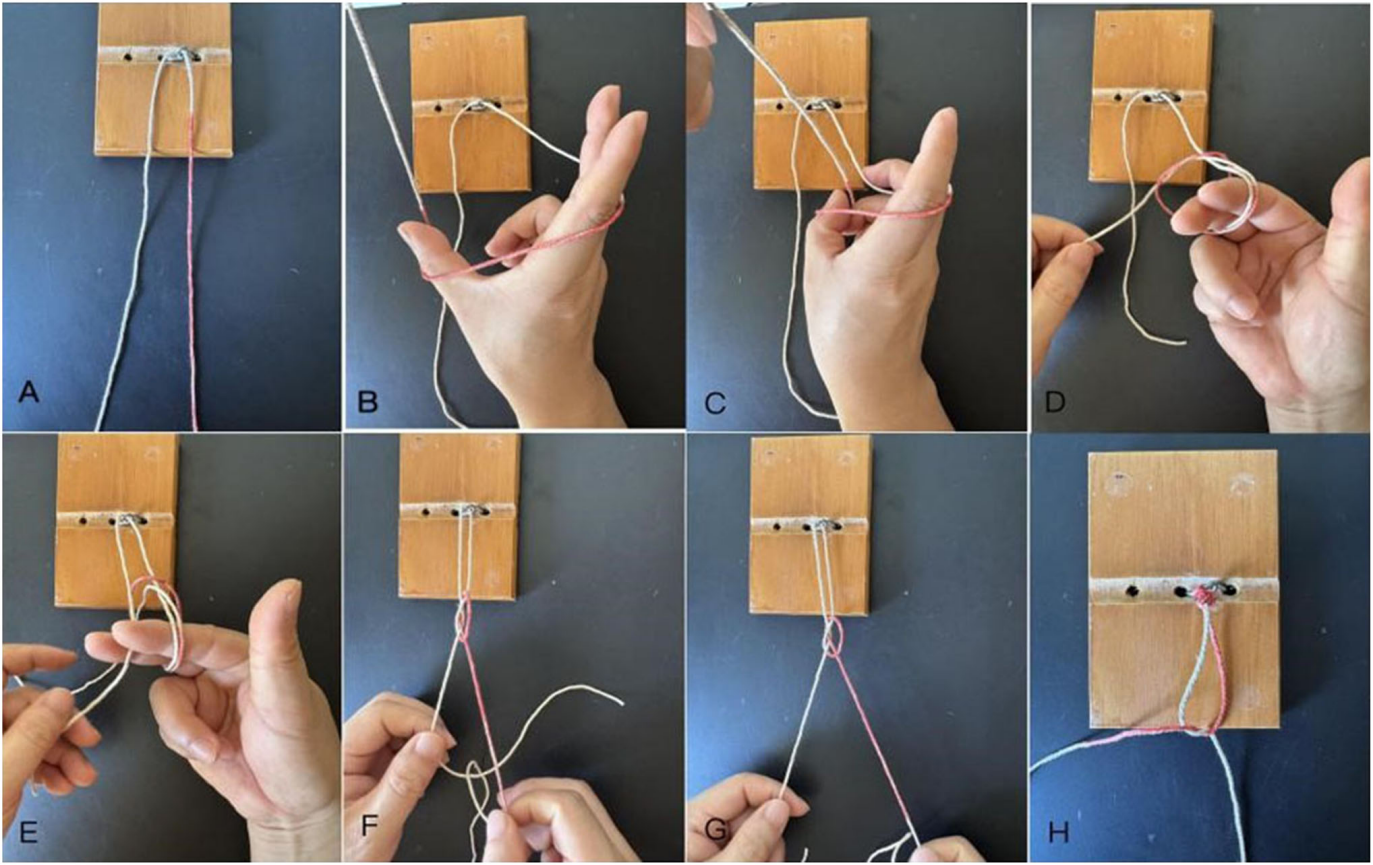
Step‐by‐step illustration of Zhang knot tying: (A) Separate suture strands; (B‐D) forming loops; (E) passing short limb through loops; (F,G) Sliding knot; and (H) final appearance of the locked Zhang knot.

1. Separate the two suture strands into a short post strand and a longer loop strand (Figure 1A).
2. Use the short limb in the left hand, form two loops with the right hand by revolving the thumb and index fingers (Figure 1B‐D).
3. Pass the end of the short limb through the two loops (Figure 1E).
4. Pull the post strand to slide the knot forward; a knot pusher may assist along the short strand if needed (Figure 1F,G).

**FIGURE 2:**
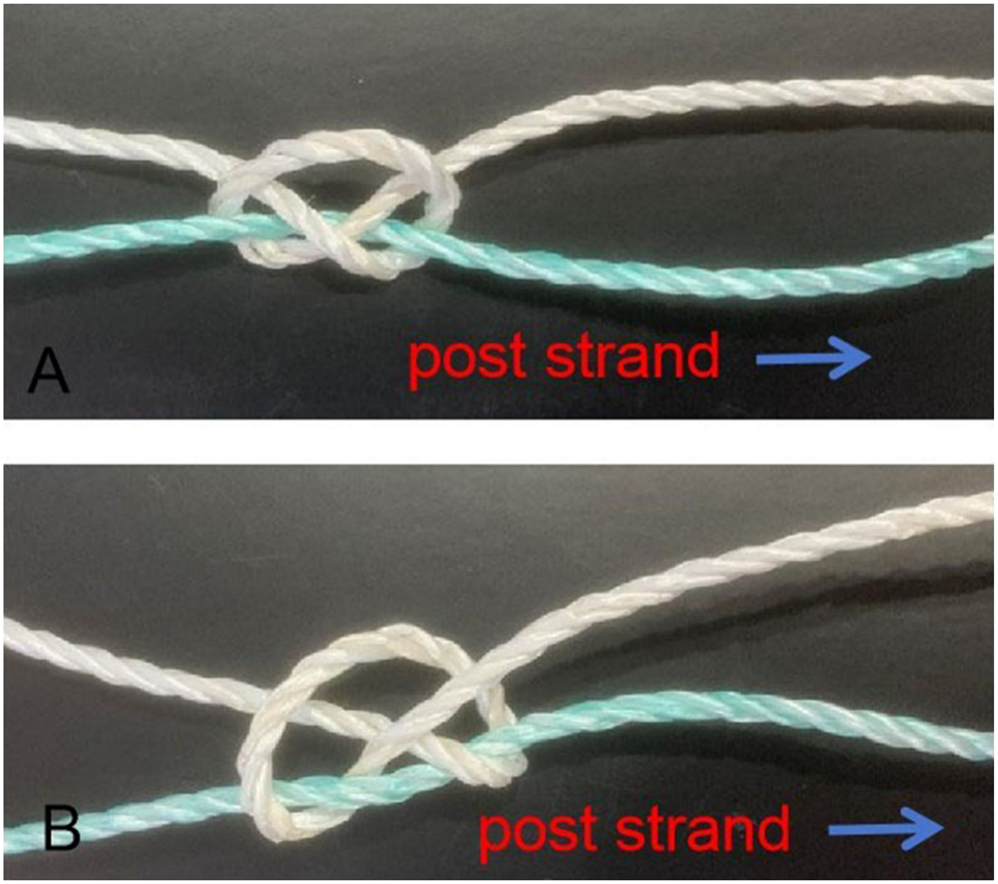
Comparison of the Zhang knot (reverse Tennessee‐type) (A) with traditional Tennessee knot (B).

At this stage, the knot resembles a reverse Tennessee knot (Figure 2A,B).

Video 1: Demonstrates the step‐by‐step procedure for tying the Zhang sliding knot.

## DISCUSSION

The Zhang knot is described as a sliding and locking configuration designed to meet the dual arthroscopic demands of high initial stability during tissue tensioning and secure final fixation.^8^, ^9^ For any new technical method, its safe and effective translation into practice hinges on a clear understanding of its execution and its inherent risks.

Successful deployment of the Zhang knot requires adherence to specific technical nuances. Advancing the sliding knot fully until it is seated directly on the target tissue is critical to minimize the risk of a loose loop that could compromise initial approximation. The key mechanistic step involves sequentially tightening the loops and then maintaining the knot pusher in a stable position while applying tension to the suture. This action reliably engages the unique suture‐interlocking mechanism. After locking the first knot, security can be reinforced with additional one‐handed knots as needed^10^, ^11^ (Table 1).

**TABLE 1:** Tips, Pearls, Pitfalls, and Risks of the Zhang Knot

Tips and Pearls
Advance the sliding knot fully until it is seated directly on the target tissue.
Sequentially tighten the loops as needed, then maintain the knot pusher in a stable position while tensioning the suture to engage the suture‐interlocking mechanism.
After locking the first arthroscopic sliding knot, additional one‐handed knots may be placed as required for security.

Pitfalls and Risks
Avoid over‐tensioning the loop limb while advancing the first half‐hitch, as this may impair smooth knot sliding.
Once the first half‐hitch is locked and the loop limb is fully tightened, the Zhang knot is definitively secured and resistant to spontaneous loosening; attempts to reverse or untie the knot at this stage may result in suture failure.

Awareness of potential pitfalls is equally vital to prevent technical failure. Surgeons must avoid over‐tensioning the loop limb while advancing the first half‐hitch, as this can impair smooth knot sliding and delivery. Most importantly, it must be emphasized that once the first half‐hitch is locked and the loop limb is fully tightened, the Zhang knot achieves a definitive and irreversible state of security. Any attempt to reverse or untie the knot at this stage risks suture damage or failure, underscoring the need for precise execution.

When executed correctly, the knot's design confers several practical advantages. Its formation—primarily involving a double‐loop maneuver with the fingers—is simple and rapidly masterable with practice. Structurally, while initiating with two half‐hitches similar to the Tennessee Slider,^12^ its defining maneuver of passing the hitch under the short limb and locking via the short loop creates a distinct, self‐reinforcing mechanism. This design translates into a low‐profile construct with minimal residual suture material upon final tightening, potentially reducing soft‐tissue irritation and conserving space in confined joints—a significant advantage in areas like the hip or the subacromial space. Furthermore, with knot pusher assistance, the locked knot exhibits excellent resistance to loosening, with security enhancing upon increased tension, thereby providing the robust initial holding strength critical for scenarios such as rotator cuff or Bankart repairs^13^ (Table 2).

**TABLE 2:** Advantages and Limitations of the Zhang Knot

Advantages
This knot primarily involves forming a double loop using the thumb, index, and middle fingers of the right hand. Surgeons can rapidly master this technique with practice
The knot configuration is simple. Upon final tightening, the amount of suture material remaining within the tissue is minimal. This reduces tissue irritation caused by retained foreign material and conserves effective space.
With the assistance of a knot pusher, once the knot is locked, it is highly resistant to loosening. The suture becomes more securely locked with increased tightening.

Limitations
This knot is applicable when used with suture anchors featuring sliding eyelets or when the suture passes freely through the tissue.
Limited biomechanical testing (unpublished data) indicates that this knot can meet satisfactory clinical needs. Further research analysis and clinical practice are required to validate its utility and operability.

We openly acknowledge the technique's limitations. Its application is contingent upon the use of suture anchors with sliding eyelets or free suture passage through tissue, restricting its universality. Furthermore, while unpublished biomechanical observations indicate that it can meet clinical demands, comprehensive comparative validation is lacking. Direct head‐to‐head biomechanical testing against established high‐strength sliding knots (e.g., Revo, SMC) under cyclic loading is necessary to objectively quantify its performance in terms of loop security, knot security, and ultimate failure load.^11^, ^14^, ^15^ The unique loop‐pulling lock also introduces a learning curve, necessitating dedicated practice in a simulated environment to achieve fluency and avoid the aforementioned pitfalls, a common phase in adopting new arthroscopic skills.

In conclusion, the Zhang knot presents a technically feasible and mechanically promising addition to the arthroscopic armamentarium. Its value lies in the combination of a learnable technique, a low‐profile final construct, and a secure locking mechanism. However, its safe application requires meticulous attention to technical details and an awareness of its irreversible locking nature. Future research must prioritize rigorous comparative biomechanical analysis and prospective clinical trials with patient‐reported outcomes to definitively establish its efficacy, optimal indications, and potential to improve upon current gold‐standard techniques.^16^

## DISCLOSURES

The authors (X.Z., Z.L., T.Z.) declare that they have no known competing financial interests or personal relationships that could have appeared to influence the work reported in this paper.
